# Identification of DEK as a potential therapeutic target for neuroendocrine prostate cancer

**DOI:** 10.18632/oncotarget.2809

**Published:** 2014-12-11

**Authors:** Dong Lin, Xin Dong, Kendric Wang, Alexander W. Wyatt, Francesco Crea, Hui Xue, Yuwei Wang, Rebecca Wu, Robert H. Bell, Anne Haegert, Sonal Brahmbhatt, Antonio Hurtado-Coll, Peter W. Gout, Ladan Fazli, Martin E. Gleave, Colin C. Collins, Yuzhuo Wang

**Affiliations:** ^1^ Vancouver Prostate Centre & Department of Urologic Sciences, University of British Columbia, Vancouver, BC V6H 3Z6, Canada; ^2^ Department of Experimental Therapeutics, BC Cancer Agency, Vancouver, BC V5Z 1L3, Canada

**Keywords:** neuroendocrine prostate cancer, patient-derived xenograft model, DEK, biomarker

## Abstract

Neuroendocrine prostate cancer (NEPC) is an aggressive subtype of prostate cancer which does not respond to hormone therapy. Research of NEPC has been hampered by a lack of clinically relevant *in vivo* models. Recently, we developed a first-in-field patient tissue-derived xenograft model of complete neuroendocrine transdifferentiation of prostate adenocarcinoma. By comparing gene expression profiles of a transplantable adenocarcinoma line (LTL331) and its NEPC subline (LTL331R), we identified DEK as a potential biomarker and therapeutic target for NEPC. In the present study, elevated DEK protein expression was observed in all NEPC xenograft models and clinical NEPC cases, as opposed to their benign counterparts (0%), hormonal naïve prostate cancer (2.45%) and castration-resistant prostate cancer (29.55%). Elevated DEK expression was found to be an independent clinical risk factor, associated with shorter disease-free survival of hormonal naïve prostate cancer patients. DEK silencing in PC-3 cells led to a marked reduction in cell proliferation, cell migration and invasion. The results suggest that DEK plays an important role in the progression of prostate cancer, especially to NEPC, and provides a potential biomarker to aid risk stratification of prostate cancer and a novel target for therapy of NEPC.

## INTRODUCTION

Prostate cancer (PCa) is the most common noncutaneous cancer and the second leading cause of death from cancer in North American men. Neuroendocrine prostate cancer (NEPC) is a highly aggressive subtype of PCa, often leading to widespread metastasis and death within months of the initial diagnosis. It is characterized by small neuroendocrine (NE)-like cells which typically express NE markers such as chromogranin A (CHGA) and synaptophysin (SYP) and do not express androgen receptor (AR) or adenocarcinoma markers, such as prostate-specific antigen (PSA) [1, 2]. Pure NEPC is rare at initial presentation and is more common in patients with a prior history of conventional PCa [3]. In recent years, accumulated biological and molecular evidence suggests that prostatic adenocarcinoma can undergo a NE transdifferentiation following androgen deprivation, and eventually progress to NEPC [4, 5]. In view of this, the emergence of more potent androgen deprivation therapies (e.g., Enzalutamide [6] and Abiraterone [7]) will likely increase the incidence of NEPC [8]. However, current treatment for NEPC provides only a marginal improvement in patients' survival. Therefore, new therapeutic targets and more effective treatments are urgently needed to improve the management of the disease.

The research of NEPC has been hampered by a lack of clinically relevant experimental *in vivo* models of the disease. Recently, we successfully developed a first-in-field model of complete neuroendocrine transdifferentiation (LTL331R) of a prostate adenocarcinoma (LTL331). The LTL331 retained histological characteristics of the original prostate adenocarcinoma of the patient, and was strongly positive for PSA and AR. Castration of mice carrying LTL331 resulted initially in a drop in tumor volume and serum PSA and gave rise to a relapsed tumor, LTL331R [9]. The latter was entirely AR and PSA negative, uniformly expressed a range of neuroendocrine markers, including SYP, CHGA, CHGB and CD56, and showed androgen-independent growth. As such, the LTL331/LTL331R xenograft model provides a valuable tool for studying mechanisms of NEPC progression and developing novel therapeutic avenues. Furthermore, both LTL331 and LTL331R xenografts exhibited very similar copy number profiles, indicating that NEPC evolves directly from adenocarcinoma cells rather than from clonal selection [4, 5]. The similar genetic profiles of LTL331 and LTL331R also suggested that in this case genetic alteration may not be the key driver for the NE transdifferentiation. Using previously obtained microarray gene expression data (GSE 41193) from LTL331 and LTL331R prostate cancer xenografts [9], we compiled a targeted, literature-driven panel of up-regulated genes (compared with adenocarcinomas) which may function as epigenetic regulators and critical factors that control the development of specific cell lineages. DEK Oncogene (DEK), a chromatin modulator, was identified as a potential therapeutic target for NEPC.

DEK belongs to a class of DNA topology modulators that was originally identified as one part of a fusion protein in a subtype of acute myeloid leukemia [10]. DEK has been shown to interact with several epigenetic modifiers, thereby serving as a hub to recruit histones, histone modifiers and chromatin remodeling factors and modulate their interaction with DNA [11–13]. Elevated expression of DEK has been detected in several types of solid tumors [14–18]. It has been suggested that DEK may promote tumorigenesis and neoplastic progression by its ability to interfere with cell division, inhibit cell differentiation, senescence and apoptosis, and to cooperate with transforming oncogenes [19–22]. However, the biological function of DEK in prostate cancer is not clear.

## RESULTS

### Elevated DEK expression in multiple NEPC xenograft models

**Figure 1:**
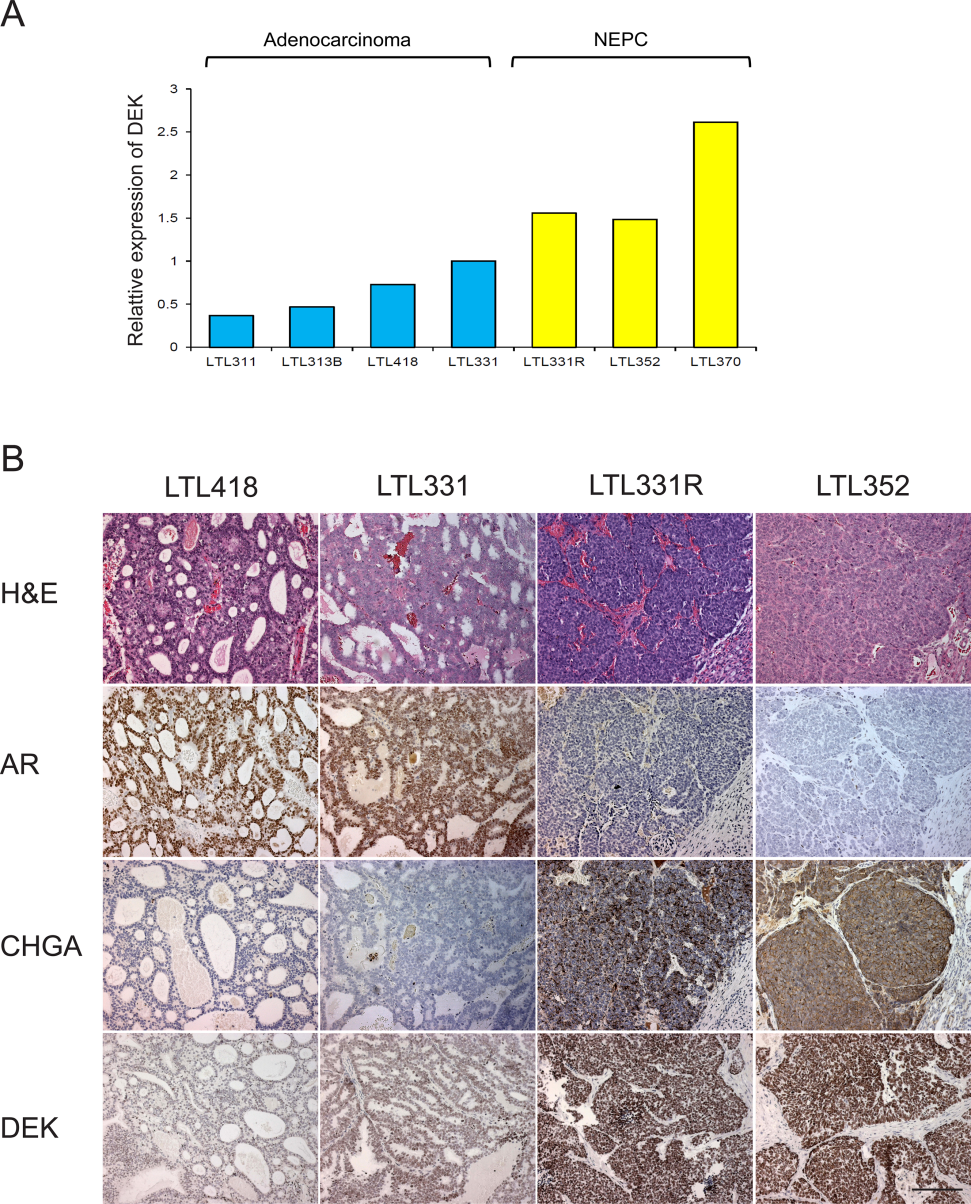
DEK expression in patient-tissue derived xenograft models **(A)** increased DEK mRNA expression is observed in NEPC xenograft models; pre-disposed increased DEK expression is observed in LTL331 compared to other adenocarcinoma xenografts, which do not give rise to NEPC after host castration; **(B)** DEK protein expression is increased in NECP xenograft models compared to adenocarcinoma models; stronger DEK staining is observed in LTL331 compared to other adenocarcinoma.

To validate the association of DEK expression with NEPC, we examined DEK mRNA expression in a panel of patient tissue-derived prostate cancer xenograft models. We observed significantly increased DEK RNA expression in multiple NEPC models (LTL331R, LTL352 and LTL370) compared to adenocarcinoma models (Fig. 1A). Among hormonal naïve prostate adenocarcinoma models, LTL331 showed higher expression of DEK compared to the other models (e.g., LTL311, LTL313B and LTL418) that did not give rise to NEPC after host castration (Fig. 1A). The protein expression of DEK was further examined by immunohistochemistry (IHC) in a panel of xenograft models. Consistent with their mRNA expression, all NEPC models showed strong nuclear staining of DEK; elevated nuclear DEK staining was also observed in LTL331 compared to other adenocarcinoma models (Fig. 1B).

### Elevated DEK expression in clinical CRPC and NEPC samples

**Figure 2:**
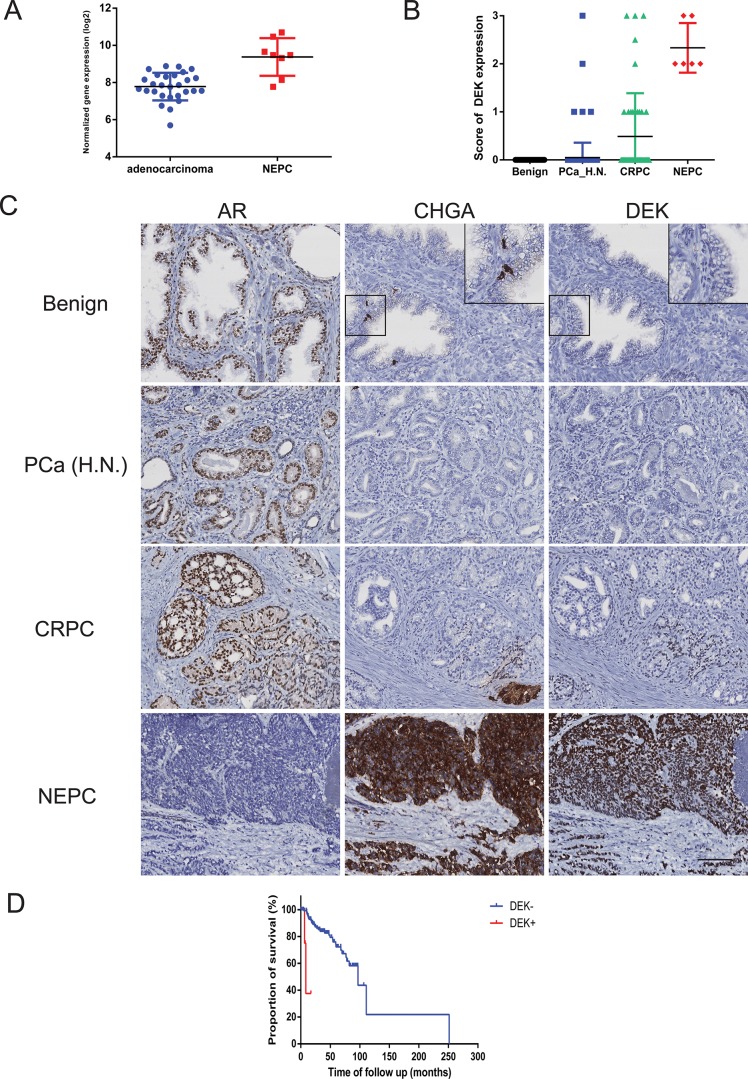
DEK expression in clinical prostate cancer samples **(A)** Elevated DEK mRNA expression is observed in clinical NEPC samples compared to adenocarcinoma; **(B)** Increased DEK protein expression is observed in clinical NEPC samples compared to CRPC, hormonal naive adenocarcinoma and benign prostate; **(C)** Representative images of AR, Chromogranin A and DEK staining in benign prostate, adenocarcinoma and NEPC. Inserts show negative DEK expression in chromogranin A positive normal NE cells; **(D)** Kaplan–Meier analysis shows that the DEK positive cases had markedly lower relapse-free survival compared with DEK negative cases (*p* < 0.001).

To determine the clinical relevance of DEK in NEPC, we mined a clinical cohort that includes 30 adenocarcinoma and 7 NEPC cases [8]. RNA-seq data of this cohort showed that DEK mRNA expression was significantly higher in NEPC tissues when compared to adenocarcinoma (*p* < 0.001, Fig. 2A). The expression of DEK protein was examined using IHC in independent clinical prostate cancer tissue microarrays (TMAs) containing 69 benign prostate, 163 adenocarcinoma, 44 CRPC and 6 NEPC cases. All the benign prostate cases and 158 out of 163 (96.93%) hormonal naïve primary adenocarcinoma cases showed negative expression of DEK protein. 4 out of 163 (2.45%) of primary hormonal naïve adenocarcinoma showed weak DEK expression and only one case showed strong DEK expression. In comparison, an increase in percentage of DEK-positive cases was observed in 13 out of 44 (29.55%) CRPC cases (*p* < 0.05). Importantly, strong to moderate nuclear DEK expression was observed in all NEPC tissues (6 out of 6, 100%), that were PSA negative and CHGA positive (Fig. 2B, C). The data suggest that increased expression of DEK is significantly associated with NEPC progression.

### Elevated DEK expression correlates with poor prognosis in prostate cancer patients

Increased DEK expression in a small proportion of hormonal naïve PCa cases and adenocarcinoma xenograft models suggests that increased DEK expression may be a pre-disposing factor in hormonal naïve cancers during NEPC progression and associated with aggressiveness. Therefore, we investigated whether increased DEK expression may provide a novel prognostic marker to aid risk stratification of hormonal naïve prostate cancers. We examined the association of DEK expression with other clinicopathological factors including age, serum PSA level, Gleason score, pathological stage and lymph node invasion status and observed a significant association of DEK expression with Gleason grade (*p* = 0.003, Table 1). For patient survival analysis, a DEK score was used to separate patients into two groups: DEK negative group (score < 1) versus DEK positive group (score > = 1). Kaplan–Meier analysis showed that the DEK-positive group had markedly shorter relapse-free survival than the DEK-negative group (*p* < 0.001, hazard ratio = 11.26, 95% confidence interval [CI]: 2.702 to 46.92, Fig. 2D). Univariate Cox regression analysis showed that Gleason grade, pathological stage, lymph node metastasis and DEK expression were independent predictors of disease free survival. Cox regression analysis of multivariates indicated that elevated DEK expression has a relative risk of 6.91 for relapse-free patients' survival (95% CI: 1.33–35.96) with a *p* = 0.022 (Table 2). It's suggested that increased expression of DEK is an independent prognostic factor in prostate cancer.

**Table 1:** Association of DEK expression with clinicopathological factors

Variables	Overall	DEK−	DEK+	*p* value
**Age**				0.2
Mean	62.08	61.95	65.93	
Median	62.69	62.68	67.8	
Range	42.55–78.79	42.55–78.79	58.22–72.12	
**PSA ng/ml**				0.908
Mean	10.84	10.86	10.13	
Median	7.43	7.47	6.45	
Range	0.53–162.00	0.53–162.00	0.60–27.00	
**Gleason score**				0.003
≤ 7	109	109	0	
≥ 8	51	46	5	
**Pathological stage**				0.202
pT1,2	82	81	1	
pT3,4	78	74	4	
**lymph node invasion status**				0.373
negative	85	83	2	
positive	27	25	2	
not done	48	47	1	

**Table 2:** Univariable and multivariable Cox regression analyses predicting disease free survival of hormonal naïve prostate cancer patients

Predictors	Univariable analysis		Multivariable analysis	
	HR (95% CI)	*p* value	HR (95% CI)	*p* value
**Pathologic Gleason**				
≤ 7	1.00 (Ref.)	-	1.00 (Ref.)	-
≥ 8	10.04 (4.3346–23.2829)	< 0.001	7.957 (2.7563–22.9727)	< 0.001
**Pathologic stage**				
pT1,2	1.00 (Ref.)	-	1.00 (Ref.)	-
pT3,4	4.855 (2.1833–10.7921)	< 0.001	1.2047 (0.4323–3.3574)	0.722
**PSA ng/ml**				
< 10	1.00 (Ref.)	-	1.00 (Ref.)	-
≥ 10	1.723 (0.8389–3.5373)	0.138	0.9989 (0.4626–2.1570)	0.998
**lymph node invasion status**				
negative	1.00 (Ref.)	-	1.00 (Ref.)	-
positive	4.9313 (2.3412–10.39)	< 0.001	2.539167 (1.15581–5.5782)	0.020
not done	0.3718 (0.1223–1.13)	0.081	0.794226 (0.21870–2.8843)	0.726
**DEK expression**				
negatie	1.00 (Ref.)	-	1.00 (Ref.)	-
positive	16.9758 (3.587–80.34)	< 0.001	6.90537 (1.32614–35.9572)	0.022

HR = hazard ratio; CI = confidence interval

### Consistently increased DEK expression during a castration time-series of LTL331

**Figure 3:**
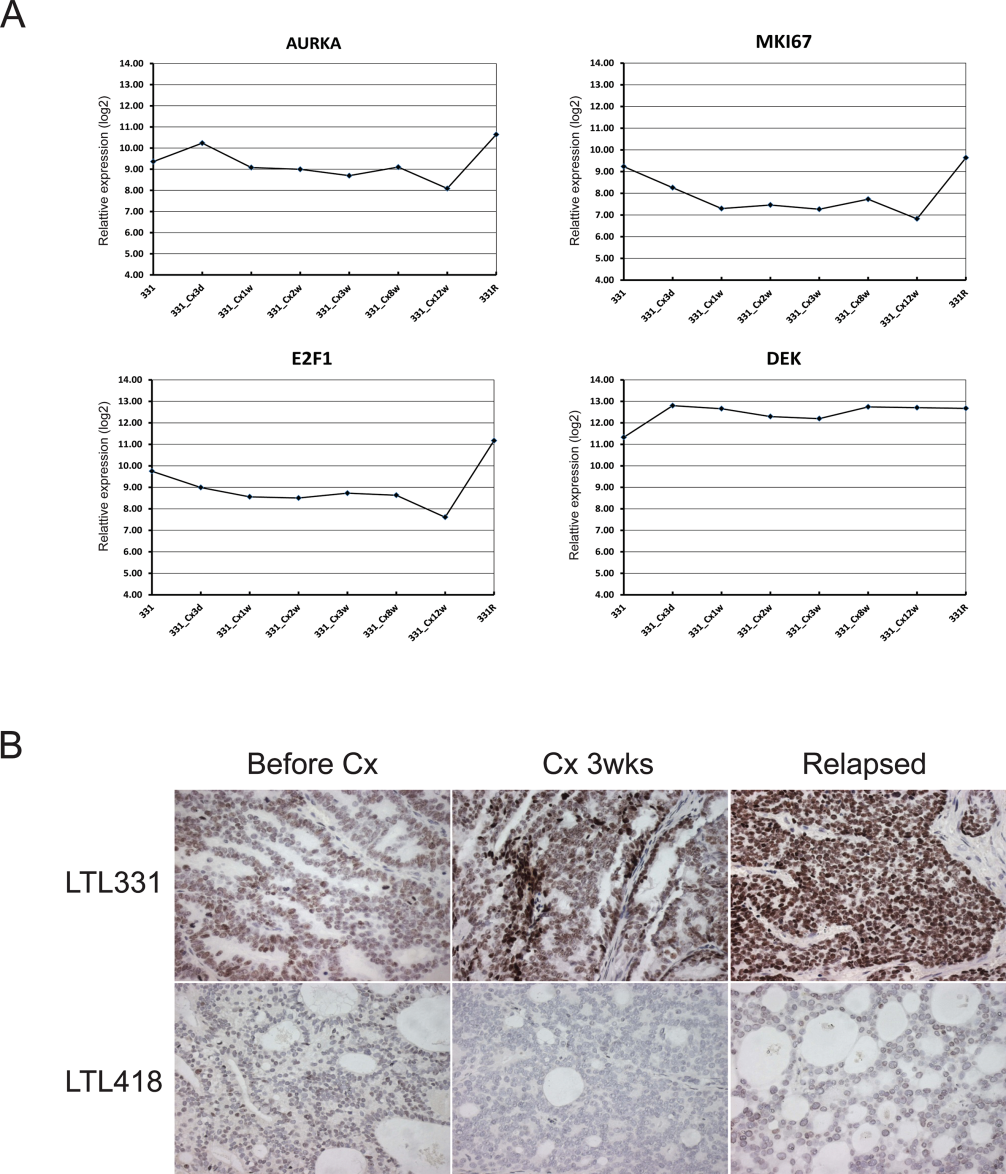
Consistent increased DEK expression during a castration time-series of LTL331 **(A)** Gene expression array analysis showed decreased expression of genes involved in cellular proliferation, cell cycle, and mitosis (i.e. MKI67, AURKA, E2F1), while increased expression was only observed in fully transformed NEPC (331R). Consistently increased RNA expression of DEK after host castration; **(B)** During the castration time-series, increased expression of DEK protein was only observed in LTL331, but not in other models, such as LTL418, which gives rise to AR positive relapsed tumor, the common form of CRPC in clinic, after host castration.

We have generated gene expression profile data from LTL331 xenografts before and after host castration at various time points (unpublished data). As expected, expression of many genes involved in cellular proliferation, cell cycle and mitosis (e.g., *MKI67*, *AURKA and E2F1)* was significantly decreased after host castration; increased expression was only detected in fully relapsed NEPC tissue. In contrast, increased DEK expression was observed after castration and stayed consistently high during the transformation to NEPC (Fig. 3A, B). Moreover, such post-castration increase in DEK expression was not observed in other adenocarcinoma models that gave rise to AR positive relapsed cancers (e.g., LTL418 and LTL313B, Fig. 3B). It is suggested that increased expression of DEK is not due to highly proliferative characteristics of NEPC, and may play an important role in the development of NEPC.

### Down-regulation of DEK suppresses prostate cancer cell growth, migration and invasion

**Figure 4:**
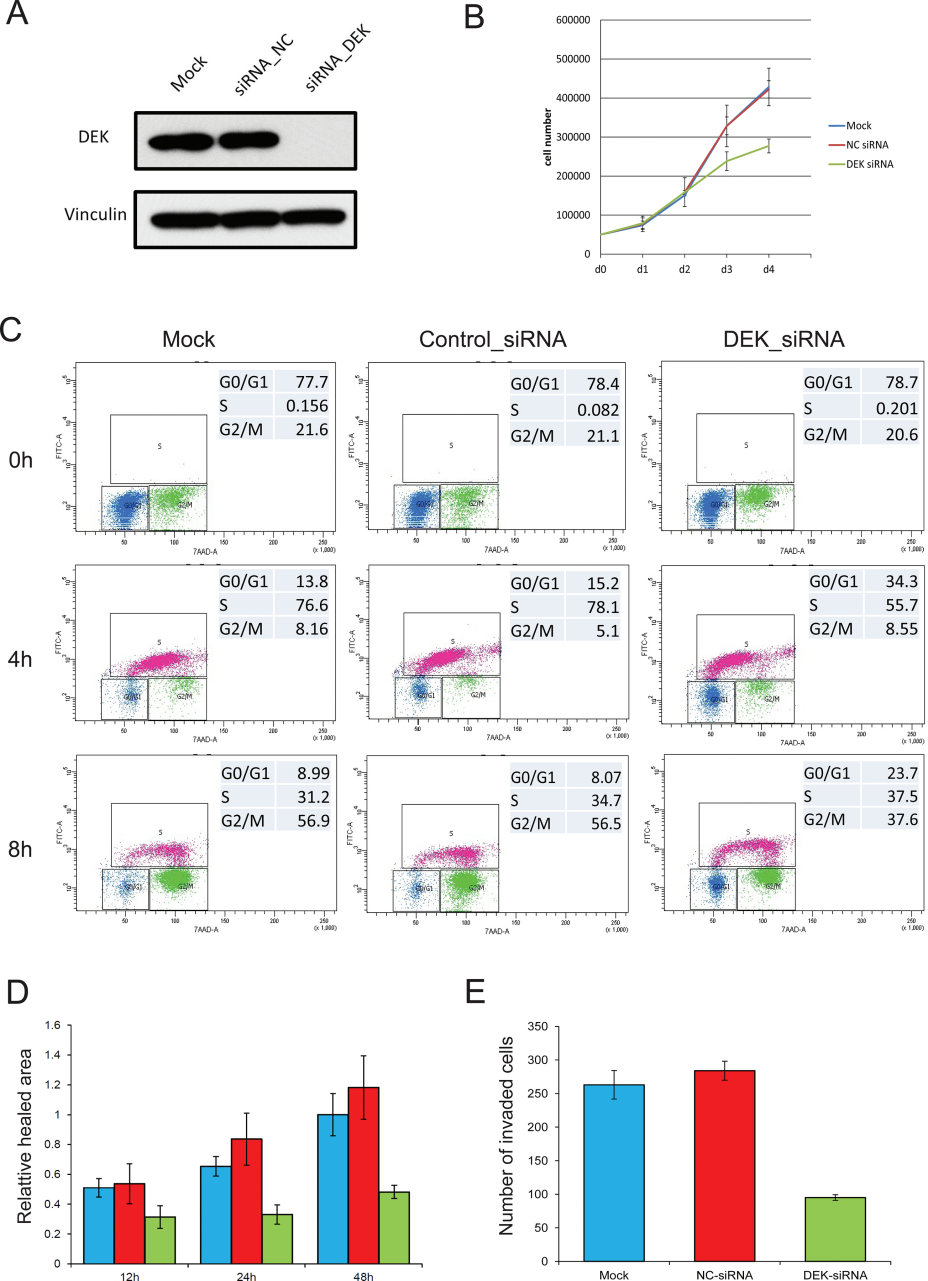
Knockdown of DEK suppresses PC-3 cell growth, migration and invasion **(A)** siRNA targeting DEK efficiently knocks down DEK expression in PC-3 cells; **(B)** Decreased DEK expression suppresses PC-3 cells growth; **(C)** Cell cycle analysis shows cell cycle was synchronized in each group by thymidine (0 h). 4 h and 8 h after release of thymidine, a significant G1/S arrest was observed in the DEK siRNA group compared to control and mock group (X axis: BrdU; Y axis: 7AAD); **(D)**. Wound healing assay showed significant suppression of cell migration in DEK knockdown cells; **(E)** Boyden chamber assay showed significant suppression of tissue invasion in DEK knockdown cells.

To study functional roles of DEK in prostate cancer, we examined effects of decreased DEK expression on PC-3 cells, a cell line characteristic of prostatic small cell neuroendocrine carcinoma [23]. A substantial loss of DEK expression in cells treated with DEK-targeting siRNA compared with those treated with vehicle or non-targeting siRNA was confirmed by Western blotting (Fig. 4A). Within 96 h after transfection, the total cell number of DEK siRNA transfected cells was significantly lower than that of control siRNA transfected and mock cells (*p* = 0.022; Fig. 4B). Cell cycle analysis showed a significant G1/S arrest in the DEK siRNA group compared to the control and mock groups after release from thymidine synchronization (*p* < 0.001; Fig. 4C). A similar G1/S arrest was also observed in DEK siRNA transfected PC-3 cells after treatment of paclitaxel (data not shown). *In vitro* scratch wound-healing and cell invasion assays were used to examine the effects of reduced DEK protein expression on migration and tissue invasion of PC-3 cells. In the wound-healing assays, DEK silencing significantly reduced PC-3 cell motility as revealed by a 48-hour wound healing assay (DEK vs Mock *p* = 0.017, DEK vs NC *p* = 0.025; Fig. 4D). In addition, Boyden chamber assays showed that DEK knockdown markedly reduced tissue invasion of the cells (DEK vs Mock *p* = 0.049, DEK vs NC *p* = 0.022; Fig. 4E).

### Potential DEK-regulated genes involved in cell proliferation and metastasis

To gain more detailed insights into the function of the DEK gene in NEPC development, we performed microarray gene expression analysis on control-treated and DEK silenced PC-3 prostate cancer cells. A total of 825 genes were found to be differentially expressed and regulated by DEK under these conditions (Student's *t*-test; FC > 1.5 and *P* < 0.05). Biological relevance of the DEK-regulated genes was further investigated by performing IPA gene function enrichment analysis. Top enrichments included gene functions related to neuronal development, proliferation of prostate cancer cells, cell cycle, cancer metastasis, and cell migration (Table 3). Listed genes belonging to these functions are likely contributors to the DEK-regulated cell proliferation and migration functions observed in our *in vitro* functional analyses.

**Table 3:** IPA function analysis of differentially expressed genes after DEK knockdown

Diseases or Functions		Predicted Activation State	Activation z-score*	*p*-Value	Molecules
**Analysis based on downregulated genes after DEK knockdown**					
*Cellular Development, Cellular Growth and Proliferation*					
	proliferation of prostate cancer cell lines	Decreased	−2.914	0.007	AKR1C3,ALCAM,CAV1,CDK6,CXCL8,FGF1,IGFBP5,LZTS1,PRKCI
	cell viability	Decreased	−2.387	0.010	ALPK2,BAG3,BCL2A1,CAMK2D,CAV1,CCNA2,CDK6,COL4A3,CXCL8,FANCA,FGF1,FPGS,ID4,IGFBP5,IL21R,MDM2,MED21,MMP1,MTMR1,NPY1R,NUPL1,PGF,POMP,PPM1A,PPP1R11,PTCH1,PTPRZ1,RRM2,SLC2A1,SUFU
	proliferation of tumor cell lines	Decreased	−2.525	0.018	AKR1C3,ALCAM,ARID3A,CAV1,CCNA2,CDCA4,CDK6,CHN2,COL4A3,CRK,CXCL8,FANCA,FGF1,GRIA1,HAS2, HIAT1,IFNAR1,IGFBP5,KISS1R,LZTS1, MDM2,MMP1,PFKFB3,PFKP,PRKCI,PTCH1,PTPRZ1,RRM2,SLC2A1,SUFU,TAGLN3,WNT7A
	cell survival	Decreased	−2.59	0.018	ALCAM,ALPK2,BAG3,BCL2A1,CAMK2D,CAV1,CCNA2,CDK6,COL4A3,CXCL8,FANCA,FGF1,FPGS,ID4,IGFBP5,IL21R,MDM2,MED21,MMP1,MTMR1,NPY1R,NUPL1,PGF,POMP,PPM1A,PPP1R11,PTCH1,PTPRZ1,RRM2,SLC2A1,SUFU
*Cellular Movement*					
	migration of cells	Decreased	−3.283	0.020	ALCAM,BAG3,BCAT1,CAMK2D,CAV1,CCNA2,CDK6,CES1,CHN2,COL4A3,CRK,CXCL8,DEK,DSG2,FCGR2A,FGF1,FZD4,HAS2,HOXD10,IFNAR1,IGFBP5,IL21R,KISS1R,LRRC15,MCF2L,MMP1,MYH10,PAQR3,PGF,PHACTR1,POU2AF1,PPM1A,PPM1F,PRKCI,PTP4A1,PTPRZ1,SDC1,SLC2A1,UTS2
	migration of tumor cell lines	Decreased	−2.953	0.001	ALCAM,CAV1,CHN2,COL4A3,CRK,CXCL8,FGF1,HAS2,IFNAR1,KISS1R,MCF2L,MMP1,MYH10,PGF,PHACTR1,PPM1F,PRKCI,PTPRZ1,SDC1,SLC2A1
*Cellular Assembly and Organization, Cellular Maintenance, Tissue Development*					
	Formation of cytoskeleton	Decreased	−2.606	0.001	CAPZB,CAV1,CDK6,CRK,CXCL8,FCGR2A,FGF1,GPR4,KISS1R,MCF2L,PPM1F,PRKCI,PSRC1
	Formation of actin filaments	Decreased	−2.744	0.002	CAV1,CDK6,CRK,CXCL8,FCGR2A,FGF1,GPR4,KISS1R,MCF2L,PPM1F,PRKCI
	Formation of filaments	Decreased	−2.606	0.001	CAPZB,CAV1,CDK6,CRK,CXCL8,FCGR2A,FGF1,GPR4,KISS1R,MCF2L,PKP1,PPM1F,PRKCI,PSRC1
	Growth of connective tissue	Decreased	−2.003	0.004	ARID3A,CAV1,CCNA2,CDK6,CXCL8,DSG2,FGF1,IFNAR1,IGFBP5,LZTS1,MCF2L,MDM2,MYH10,NPR3,PAQR3,PGF,PTCH1,PTPRZ1,UTS2
*Cell transformation*					
	Cell transformation	Decreased	−2.675	0.004	ASPH,BCL2A1,CAV1,CCNA2,CDK6,CRK,FGF1,GPR4,HAS2,MCF2L,MDM2,PRDX3,PRKCI,PTCH1,WNT7A
**Analysis based on upregualated genes after DEK knockdown**					
*Cell Cycle*					
	S phase of tumor cell lines	Decreased	−2.401	0.021	CCNG2,CDH1,ESR2,IL1B,IL6,MXD4,PMEPA1
*Nervous System Development and Function*					
	Morphology of nervous system	Decreased	−2.207	0.003	ASIC3,BMPR1B,CELSR3,COL13A1,COLQ,CTSF,DKK1,EGR2,ESR2,GFI1,GJC2,GLDN,HESX1,HHAT,HOXD3,ID1,IL1B,IL6,IL6ST,IRX6,KCND2,KCNQ1,LAMC1,LRRK2,MAGI2,MAPK8IP2,NAB1,NFATC4,NRP1,NTF4,POU4F1,PROS1,RELN,SALL4,SH2D3C,SLC12A5,SNCA,THRA,UBE4B,UCN

* Positive and negative Z-score cutoffs are ≥ 2.0 and ≤ −2.0, respectively.

## DISCUSSION

NEPC is a lethal form of AR negative prostate cancer with most patients dying within 2 years of diagnosis despite very aggressive chemotherapeutic regimens [24–26]. This aggressive variant of prostate cancer has been increasingly recognized in the clinic. However, molecular alterations in NEPC remain largely unknown. Recently we successfully developed a first-in-field complete NE transdifferentiation xenograft model. Comparative analysis of gene profiling data of parental adenocarcinoma (LTL331) and relapsed NEPC (LTL331R) showed increased expression of a panel of NE markers in the NEPC model, which is consistent with the findings in clinical NEPCs. It is suggested that this model provides a valuable tool for identifying key molecules involved in NEPC development.

In this study, we showed for the first time that DEK, a chromatin binding protein, is abnormally expressed in NEPC clinical samples and xenograft models and is associated with neuroendocrine differentiation. Interestingly, the increase of DEK expression was only observed in LTL331 but not in other adenocarcinoma models that do not give rise to NEPC after host castration. Consistently, we observed increased DEK expression in only 2.45% of hormonal naive PCa cases and an association of increased DEK expression with poor clinical outcome. These data indicate that the increase of DEK expression may predispose for NEPC development. Retrospective examination of DEK expression in hormone-naïve tissue specimens from patients who have or have not developed NEPC will further validate this hypothesis.

There is an urgent need to identify markers to aid risk stratification of hormone-naïve prostate cancer. In this study, we demonstrated that increased DEK expression is an independent prognostic factor in prostate cancer. It should be noted that unlike other NE markers, DEK expression was not observed in non-proliferative, normal NE cells, which indicates that DEK expression is not just a reflection of the NE phenotype. It is suggested that increased expression of DEK may be associated with the aggressiveness of PCa and provides improved prognostic information alone or in combination with other NE markers. Evaluation using independent patient cohorts will be helpful to determine the veracity of the findings.

It remains unclear why DEK expression increased during the development of NEPC. It has been reported that DEK can be transcriptionally activated by the E7 oncogene, a classical negative regulator of the retinoblastoma (Rb) protein in human papillomavirus (HPV)-positive cervical cancer cells and primary human keratinocytes [27, 28]. Moreover, E2F1 was shown to be able to bind to the DEK promoter and involve an activation of DEK transcription [29]. By examination of RNA expression of LTL331 before and a series of time points after host castration, we observed decreased expression of E2F1 in series of post-castration samples, whereas increased expression was only detected at the last time point, i.e. fully transformed NEPC. Conversely, consistently increased DEK RNA and protein expression was observed at all the castration time points, suggesting that another mechanism beyond E2F1 is involved in the regulation of DEK expression. Since increased DEK expression was observed immediately after host castration, it is possible that DEK is negatively regulated by AR signaling in adenocarcinoma. However, this hypothesis was not supported by the lack of DEK post-castration up-regulation in other adenocarcinoma xenograft models (such as LTL313B and LTL418). Investigating the mechanisms of regulation in DEK expression will be an avenue for future research directions.

The functional role of DEK in prostate cancer is not clear. In this study, we observed a significant suppression of cell proliferation and cell invasive ability by DEK silencing. Cell cycle analysis demonstrated that the suppression of cell proliferation was due to a G1/S arrest. These findings are consistent with an oncogenic role of DEK previously reported in other types of cancer [30, 31]. Further comparative gene expression profiling analysis of DEK-knockdown and control PC-3 cells demonstrated that a number of genes involved in the cell cycle and cancer metastasis were differentially expressed after DEK knockdown. These potential DEK-regulated genes are likely contributors to the DEK-dependent cell proliferation and migration functions observed in *in vitro* functional analyses. We also observed a significant decrease in the expression of some WNT ligands (WNT7A and WNT7B) in DEK silenced PC-3 cells, which is consistent with the findings of a recent study in breast cancer that DEK can drive the proliferation of cells through stimulated expression and secretion of Wnt ligands [31]. It suggested that the interaction of DEK and WNT pathways may play an important role in multiple types of cancer. Interestingly, a number of genes involved in neural system development were enriched in the IPA function analysis in our study (Table 3). Further study on DEK regulated genes/pathways will provide insights to the mechanism of DEK regulated NE differentiation of prostate cancer. Recently, DEK was identified as an SPOP substrate that exhibited decreases in ubiquitylation and proteasomal degradation and increase in expression in SPOP-mutant prostate cancer [32]. This study showed that in LHMAR and LNCaP cell lines, SPOP mutant-related or vector-based overexpression of DEK significantly promotes cell invasion and knockdown of DEK decreases cell invasion of cells overexpressing mutant SPOP, which implicates DEK as an oncogenic effector in prostate cancer. This study provides further independent evidence for the functional role of DEK in prostate cancer.

Currently, there is no effective targeted therapy for NEPC patients. New targeted agents in the preclinical and clinical development stage, such as an AURKA inhibitor, PHA-739358, showed promising effects [8]. AURKA is known to play a role in mitosis. Interestingly, in the process of NE transdifferentiation of LTL331/LTL331R, we observed that a number of genes associated with cell proliferation and mitosis (e.g., *MKI67*, *AURKA and E2F1*) was significantly decreased at multiple time points after host castration and increased only in fully relapsed NEPC tissue. This suggests that increased expression of these genes in NEPC is likely a reflection of highly proliferative characteristics of NEPC rather than key regulators of NEPC development. On the contrary, we observed (1) consistently increased DEK expression in post-castration LTL331, multiple NEPC models and clinical NEPC samples and (2) significant suppression of cell proliferation and migration in DEK-depleted cancer cells.

In conclusion, the findings of the present study suggest that DEK plays an important role in the progression of prostate cancer, especially to NEPC. Elevated DEK protein expression may serve as a novel prognostic factor for prostate cancer patients. The DEK gene may represent a new target for therapy of NEPC.

## MATERIALS AND METHODS

### Materials and animals

Chemicals, solvents, and solutions were obtained from Sigma-Aldrich Canada Ltd, Oakville, ON, Canada, unless otherwise indicated. Six- to eight-week old NOD/SCID IL2 receptor gamma chain null (NSG) mice were bred by the BC Cancer Research Centre Animal Resource Centre, BC Cancer Agency, Vancouver, Canada.

### Xenografts

The patient-derived prostate cancer adenocarcinoma tissue lines were maintained via serial transplantation of subrenal capsule xenografts in male NSG mice supplemented with testosterone, as previously described [33]. NEPC xenografts LTL352, LTL370 were maintained in intact male NSG. To determine the response of LTL331 to castration, the testosterone pellet was removed and mice were castrated after tumor volume reached > 200 mm^3^. Animal care and experiments were carried out in accordance with the guidelines of the Canadian Council on Animal Care.

### Clinical prostate cancer tissues

The prostate cancer specimens (69 benign prostate, 163 adenocarcinoma, 44 CRPC and 6 NEPC cases) were obtained from the Vancouver Prostate Centre Tissue Bank. Specimens were obtained from patients, with their informed consent, following a protocol approved by the Clinical Research Ethics Board of the University of British Columbia (UBC) and the BC Cancer Agency. Tissue microarrays (TMAs) were constructed [34] at the Prostate Centre, Vancouver General Hospital (VGH). The H&E slides were reviewed and the desired areas were marked on them and their correspondent paraffin blocks. TMAs were manually constructed (Beecher Instruments, MD, USA) by punching duplicate cores of 1 mm for each sample.

### Post-operative follow-up

Following surgery, patients were tested every 6 months for serum PSA levels. PSA recurrence was defined as a sustained elevation, on two or more occasions, of serum total PSA > 0.2 ng/ml and was assigned to the date of the first elevated value.

### Immunohistochemistry

Immunohistochemical staining with monoclonal mouse antibody against DEK (BD Biosciences, San Jose, CA, USA) was conducted using a Ventana autostainer (model Discover XT; Ventana Medical System, Tucson, AZ) with an enzyme-labelled biotin-streptavidin system and a solvent-resistant DAB Map kit (Ventana). All sections used for immunohistochemistry were counterstained with 5% (w/v) Harris hematoxylin.

### DEK protein staining scoring

DEK nuclear staining of tissues was evaluated by two pathologists and given a score of 0, 1, 2 or 3, representing no DEK staining, weak, moderate and strong DEK staining intensity, respectively.

### Cell cultures

Human PC-3 prostate cancer cell lines were obtained from the American Type Culture Collection (ATCC, Manassas, VA). Cultures were maintained in RPMI-1640 medium supplemented with 10% fetal bovine serum.

### siRNA transfection

Small interfering RNA (siRNA) targeting DEK (siDEK) and negative control (scrambled) siRNAs were purchased from Dharmacon (Cat. No. L-003881-00 and D001810-10, Chicago, IL). Cells were transfected with 20 nM siRNA in oligofectamine reagent (Invitrogen, Carlsbad, CA) following the manufacturer's instructions.

### Western blotting

PC-3 cell lysates were prepared using cell lysis buffer supplemented with a protease inhibitor cocktail (Roche, Basel, Switzerland); total lysate protein was determined using the BCA protein assay (Pierce, Rockford, IL). For Western blotting, typically 10 μg whole cell lysate was run on 8% SDS polyacrylamide gel. The samples were electrotransferred to PVDF membrane and nonspecific binding was blocked in TBST buffer containing 5% bovine serum albumin. The chemoluminescent signal was detected using SuperSignal West Femto Maximum Sensitivity Substrate (Pierce). The following antibodies were used: anti-DEK (BD Biosciences), anti-vinculin (Sigma-Aldrich).

### Wound healing cell migration assay

PC-3 cells were seeded into 6-well culture plates using their regular maintenance medium. After the cells had reached confluence, the medium was removed and a plastic pipette tip was drawn across the center sections of the wells to produce clean ~1-mm-wide wounds in the monolayers. 0.5 μM mitomycin C was added to the culture medium after wounds had been made. Images were taken immediately after generating the wound, and after 12, 24 and 48 hours of incubation. The cell-recovered areas were measured to estimate the extent of cell migration [35]. The average percent wound healing was determined based on 3 measurements of the wound area and were expressed as means ± SD. Statistical significance was established using the Student's t-test.

### Modified boyden chamber assays

Tumor cell invasion assays were performed using modified Boyden chambers consisting of 8 μm pore filter inserts in 24-well plates (BD Biosciences, San Jose, CA) as described elsewhere [35]. PC-3 cells were trypsinized and resuspended in serum-free RPMI-1640 medium and plated on Matrigel-coated membranes of the upper compartments. Cells were incubated at 37°C for 22 hours in a CO_2_ incubator, using 5% fetal bovine serum in the lower chambers as a chemoattractant. Following incubation, the inserts were pulled out and the non-invading cells on the upper surface were removed with a cotton swab. The cells on the lower surface of the membrane were fixed in 4% paraformaldehyde, air-dried and stained with DAPI VECTASHIELD solution (Vector Laboratories Inc., Burlingame, CA). Membranes were scanned using a Zeiss AxioPlan 2 fluorescent microscope. The number of invaded cells was counted. Results were expressed as means ± SD. Statistical significance was established using the Student's *t*-test.

### Total RNA isolation and quantitative Real-Time PCR (qRT-PCR)

Total RNA was isolated from cultured cells using the RNeasy mini kit (Qiagen Inc., Hilden, Germany) following the manufacturer's instructions. Total RNA (1 μg) was used to synthesize cDNAs using a QuantiTect Reverse Transcription Kit (Qiagen Inc.). qRT-PCR reactions using KAPA SYBR Fast Universal (Kapa Biosystems, Woburn, MA) were performed in a ViiA 7 Real-Time PCR system (Applied Biosystems, Foster City, CA). The primer sequences used are *DEK*, forward 5′-GCCGAAATCCGCGGTTCA-3′ and reverse 5′-CTCTCTCTGTAAGGAAGAGACTTGC-3′; *GAPDH,* forward 5′-CACCAGGGCTGCTTTTAACTC-3′ and reverse 5′-GACAAGCTTCCCGTTCTCAG-3′.

### Microarray analysis

RNA for microarray analysis was extracted from two replicates of siDEK knockdown and siControl treated PC-3 cells. RNA sample quality was assessed with the Agilent 2100 Bioanalyzer and NanoDrop ND-2000 UV-VIS spectrophotometer, such that only samples with RNA Integrity Number (RIN) ≥ 8.0, A260/280 OD values between 1.8 and 2.0, and an A260/A230 OD value of 2.0 were used for one-color labelling using Agilent's One-Colour Microarray-Based Gene Expression Analysis Low Input Quick Amp Labelling v6.0 (Agilent Technologies, Santa Clara, CA). 100 ng of total RNA was used to generate cyanine-3-labelled cRNA. RNA from samples were hybridized on Agilent SurePrint G3 Human GE 8×60K Microarray v2 (Design ID 039494). Arrays were scanned with an Agilent DNA Microarray Scanner at a 3 μm scan resolution and image data were processed with Agilent Feature Extraction 11.0.1.1. Processed signals were quantile normalized with Agilent GeneSpring 12.0. Raw expression data have been deposited in the NCBI's Gene Expression Omnibus and are accessible through http://www.ncbi.nlm.nih.gov/geo/query/acc.cgi?token=erqxugakzjmrrwp&acc=GSE61214.

### Biostatistical or bioinformatics analysis

Microarray gene expression data were filtered to improve data quality prior to downstream analysis. Only probes with gene annotations and detectable expression levels (greater than 3 in log2 scale) were retained. Differentially expressed genes induced by DEK knockdown were identified as genes with > 1.5 fold difference in the siDEK-treated samples relative to the control samples and with *p*-value < 0.05 (Student's *t*-test). Significantly differentially expressed genes were interpreted for functional gene enrichments using the Ingenuity Pathway Analysis software (IPA; June 2014 release). Statistical over-representation of functions was calculated using the Fischer's exact test and Benjamini-Hochberg (BH) multiple-test correction method.

### Statistical analysis

The *t*-test was used to compare mean DEK protein expressions between each pathological group. The Kaplan–Meier method was used to estimate curves for relapse-free survival and comparisons were made with the use of the log-rank test. Hazard ratios were calculated using Cox proportional hazard models. The Student's t test was used for comparison of *in vitro* studies. All tests of significance were two sided, and differences were considered statistically significant with *p* values less than 0.05.
